# Effects of Li^+^ conduction on the capacity utilization of cathodes in all-solid-state lithium batteries

**DOI:** 10.3389/fchem.2023.1169896

**Published:** 2023-04-21

**Authors:** Zhiping Wang, Shipai Song, Chunzhi Jiang, Yongmin Wu, Yong Xiang, Xiaokun Zhang

**Affiliations:** ^1^ School of Materials and Energy, University of Electronic Science and Technology of China, Chengdu, Sichuan, China; ^2^ State Key Laboratory of Space Power-sources Technology, Shanghai Institute of Space Power-sources, Shanghai, China; ^3^ Advanced Energy Research Institute, University of Electronic Science and Technology of China, Chengdu, Sichuan, China; ^4^ Sichuan Provincial Engineering Research Center of Flexible Display Material Genome, University of Electronic Science and Technology of China, Chengdu, Sichuan, China

**Keywords:** all-solid-state lithium batteries, cathode, capacity, Li^+^ diffusivity, modeling

## Abstract

Li^+^ conduction in all-solid-state lithium batteries is limited compared with that in lithium-ion batteries based on liquid electrolytes because of the lack of an infiltrative network for Li^+^ transportation. Especially for the cathode, the practically available capacity is constrained due to the limited Li^+^ diffusivity. In this study, all-solid-state thin-film lithium batteries based on LiCoO_2_ thin films with varying thicknesses were fabricated and tested. To guide the cathode material development and cell design of all-solid-state lithium batteries, a one-dimensional model was utilized to explore the characteristic size for a cathode with varying Li^+^ diffusivity that would not constrain the available capacity. The results indicated that the available capacity of cathode materials was only 65.6% of the expected value when the area capacity was as high as 1.2 mAh/cm2. The uneven Li distribution in cathode thin films owing to the restricted Li+ diffusivity was revealed. The characteristic size for a cathode with varying Li^+^ diffusivity that would not constrain the available capacity was explored to guide the cathode material development and cell design of all-solid-state lithium batteries.

## 1 Introduction

Lithium-ion batteries have been widely applied in consumer electronics, electric vehicles, and smart grids (Hannan et al., 2018; Li et al., 2018; Zubi et al., 2018). However, the flammable liquid electrolytes induce frightening safety problems (Wang et al., 2012; Wang et al., 2018; Duan et al., 2020). In addition, the lithium dendrite growth in liquid electrolytes impedes the use of lithium metal anodes and restricts lithium-based batteries to low energy densities (Liu and Lu, 2017; Kong et al., 2018; Wang T et al., 2020). All-solid-state lithium batteries (ASSLBs), which employ solid electrolytes and metallic lithium anodes, hold great promise for developing the next generation of energy storage technologies with high energy density and safety (Manthiram et al., 2017; Gao et al., 2018; Randau et al., 2020).

In the past decades, the R&D on solid electrolyte technologies has made considerable strides in the aspects of materials, process, equipment, and compatibility with lithium metal anodes (Xia et al., 2019; Miao et al., 2020; Peng et al., 2020; Zhu et al., 2020; Guan et al., 2021; Lu et al., 2021; Mou et al., 2021; Song et al., 2022; Wei et al., 2023a). Meanwhile, the cathode has been the major bottleneck for achieving ASSLBs with high energy densities (Chen et al., 2017; Judez et al., 2018; Ma, 2018). The Li^+^ conduction in the cathode of ASSLBs is limited due to the lack of an infiltrative network for Li^+^ transportation, compared with the lithium-ion batteries based on liquid electrolytes. The capacity utilization ratio of the cathode in ASSLBs, which is the ratio of practically available capacity to the theoretical expectation, decreases as the particle size and/or thickness of the cathode increases. Although the Li^+^ diffusivity of cathode materials could be improved *via* element doping and surface coating (Mou et al., 2021; Wei et al., 2023b; Liu et al., 2018; Song et al., 2020), it is necessary to determine the characteristic size for cathode materials with different Li^+^ diffusivities that would not impair the capacity utilization rate (CU). The determined characteristic sizes would help design the diameter of cathode particles or the thickness of cathode thin films and promote the development of advanced cathode technologies.

In this work, all-solid-state, thin-film lithium batteries (ASSTFLBs), with the cell structure of LiCoO_2_-LiPON-Li (Supplementary Figure S1), were chosen as the model system to investigate the characteristic sizes of cathode because the thickness of LiCoO_2_ thin films can be easily controlled in the experimental and modeling studies. ASSTFLBs based on LiCoO_2_ thin films with different thicknesses were fabricated and tested. An area capacity of 1.2 mAh/cm^2^ was achieved when the thickness of LiCoO_2_ thin film was 25.7 μm. To the best of our knowledge, this is the highest value yet reported for ASSTFLBs. However, the CU decreased from 99.2% to 65.6% as the thickness of LiCoO_2_ thin films increased from 1.21 to 25.7 μm. To analyze the bottleneck factors that affect the CU, a one-dimensional (1D) model of ASSTFLBs was established. The simulations showed that the ASSTFLBs with thicker cathodes possess higher solid-phase lithium concentration (SPLC) after Li extraction, and the uneven distribution of the SPLC also intensifies as the LiCoO_2_ thickness increases. Additionally, the increased Li^+^ diffusivity effectively reduces the SPLC in thick LiCoO_2_ thin films after Li extraction. Thus, the constrained CU should be mainly attributed to the limited Li^+^ conduction in the cathode. Finally, the quantitative relationship between the CU of the cathode thin film and its thickness is calculated for cathodes with an assumed Li^+^ diffusivity of 1 × 10^−15^, 10^−14^, and 10^−13^ m^2^/s, respectively.

## 2 Experiment and modeling

### 2.1 Fabrication of ASSTFLBs

**FIGURE 1:**
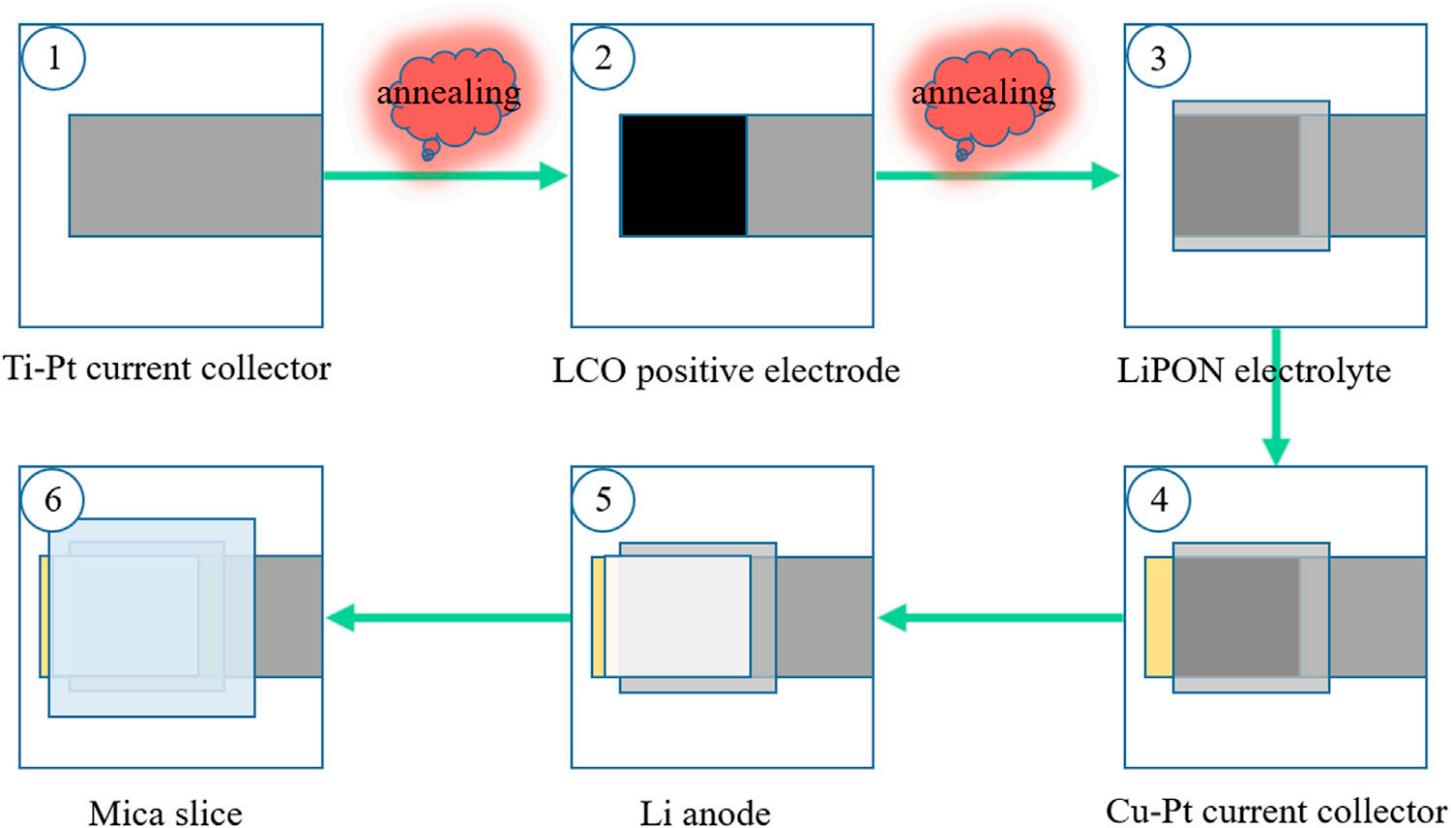
Schematic illustration of ASSTFLB fabrication.

ASSTFLBs with the structure of (Ti-Pt)-LiCoO_2_-LiPON-Li-(Cu-Pt) were fabricated on glass substrates *via* physical vapor depositions analogous to the method reported in Donders et al. (2013) and Song et al. (2010) (Figure 1). First, a metallic titanium (Ti) thin film with a thickness of 20 nm was deposited on a glass substrate *via* DC sputtering, followed by a metallic platinum (Pt) thin film with a thickness of 100 nm. The deposited double-layered thin film was then annealed at 400 °C for 30 min. Afterward, the temperature of the annealing chamber was controlled to decrease to room temperature linearly within 4 h. Second, a LiCoO_2_ thin film was deposited on the Ti-Pt current collector (CC) *via* sputtering supplied by RF and DC hybrid power. By controlling the sputtering time, LiCoO_2_ thin films with thicknesses of 1.21, 2.56, 10.17, and 25.70 μm were obtained. The samples were heated to 500 °C with a ramp rate of 300 °C/h and annealed at 500 °C for 900 min. Then, the samples were linearly cooled to room temperature within 7 h. Third, a ∼2-μm thin film of LiPON electrolyte was sputter-deposited on LiCoO_2_ using the RF and DC hybrid power supply. Fourth, 20 nm of copper (Cu), followed by 100 nm of platinum (Pt), was sputter-deposited using a DC power supply to fabricate the Cu-Pt CC for the Li metal anode. Fifth, 2 μm of lithium metal was deposited using an evaporator in a dry room with a dew point of −50 °C. Sixth, UV glue was applied to the ASSTFBs before they were covered with mica sheets. The packaged cells were cured for 5 min under a UV lamp in the dry room. The footprint of the fabricated ASSTFLBs is 1.2 × 1.2 cm^2^.

### 2.2 Characterization and test

The thickness of LiCoO_2_ thin films was determined using cross-sectional images obtained by scanning electron microscopy (SEM, Zeiss Sigma 300) at 10 kV. The cycling performance tests of the ASSTFLBs were performed between 2.7 and 4.2 V using vs. Li^+^/Li with varied charge–discharge rates at room temperature by the battery test equipment (NEWARE CT-3008). According to the theoretical specific capacity of LiCoO_2_ (149 mAh/g for the cutoff voltage from 2.7 to 4.2 vs. Li^+^/Li), ASSTFLBs with a cathode of 1.21, 2.56, and 10.17 μm were first cycled at a low rate of 0.1C for three laps, and then cycled at 0.2, 0.5, 1, 2, and 5C for one lap, and finally charged and discharged at 10 C. The ASSTFLBs with the 25.70-μm cathodes were tested at a rate of 0.05C for charge and discharge cycles. The expected capacities for fabricated ASSTFLBs with cathodes of 1.21, 2.56, 10.17, and 25.70 μm LiCoO_2_ are 0.08, 0.18, 0.69, and 1.18 mAh/cm^2^, respectively. The CU was determined by dividing the measured or calculated capacities of the LiCoO_2_ cathode by the theoretically expected values. The theoretical capacities of experimental ASSTFLBs are provided in Supplementary Table S3.

### 2.3 Modeling of ASSTFLBs

A 1D model of ASSTFLBs with a 2-μm lithium metal anode, a 2-μm LiPON electrolyte, and a LiCoO_2_ cathode with different thicknesses (5, 10, 15, 20, 25, and 30 μm) was constructed using COMSOL Multiphysics software (Ramadesigan et al., 2012; Kazemi et al., 2019; Geng et al., 2021). The key parameters and their values in the presented model are summarized in Supplementary Table S1. The Li^+^ diffusivity of the LiCoO_2_ thin film was assumed to be 1 × 10^−15^ (D15), 1 × 10^−14^ (D14), and 1 × 10^−13^ m^2^/s (D13), based on the progress of cathode material research (Wang X et al., 2020; Mou et al., 2021). The charge–discharge curves and SPLC of LiCoO_2_ in the modeled ASSTFLBs were calculated.

The electrode reaction is described by the Butler–Volmer equation:

$$i=i_{a}-i_{c}=Fk\left\{C_{0}\left(0,t\right)e^{\left[-\frac{\alpha F\eta }{RT}\right]}-C_{R}\left(0,t\right)e^{\left[-\frac{\left(1-\alpha \right)F\eta }{RT}\right]}\right\},$$ (1)

where *i_a_* is the anodic current, *i_c_* is the cathodic current, F is the Faraday constant, k is the reaction rate constant, *C_0_* is the concentration of the species, e is the natural constant, α is the charge transfer coefficient of the reaction, R is the molar gas constant, T is the temperature, and *η* is overpotential (Danilov et al., 2011; Raijmakers et al., 2020).

The mass transfer process in a solid is described by the Nernst–Planck equation:

$$N_{i}=-D_{i}\nabla c_{i}-z_{i}u_{m,i}Fc_{i}D_{i}\nabla \emptyset +c_{i}u,$$ (2)

where *D_i_* is the diffusion coefficient (m^2^/s), ∇*c_i_* is the ion concentration gradient (mol/cm^3^), Z*_i_* is the charge of substance, *u_m,i_* is the mobility (s.mol/kg), ∇*∅* represents the potential gradient, and *u* represents the velocity vector (m/s) (Doyle et al., 1993; Fuller et al., 1994).

## 3 Results and discussion

**FIGURE 2:**
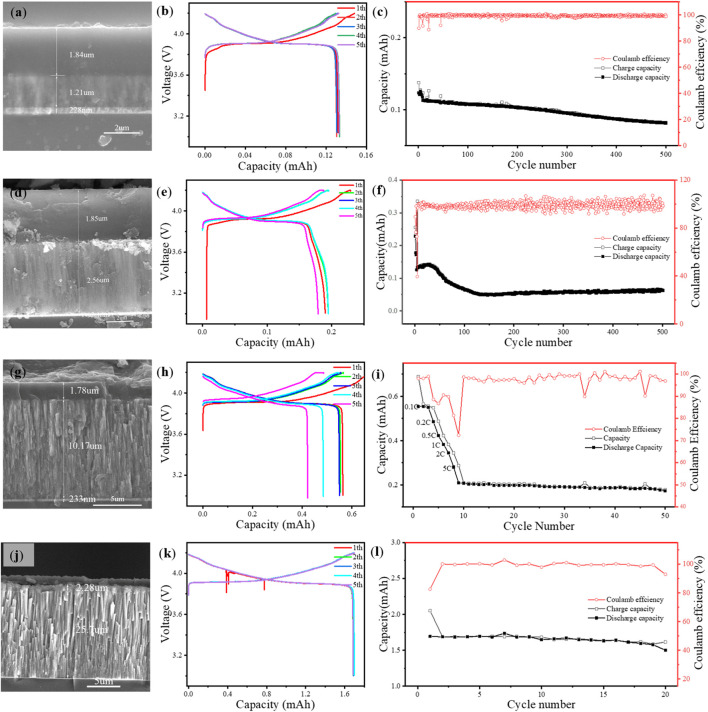
Cross-sectional SEM images, charge and discharge curves of first five cycles, and cycling performance of ASSTFLBs with varying thicknesses of LiCoO_2_ thin film: 1.21 μm **(A–C)**, 2.56 μm **(D–F)**, 10.17 **(G–I)**, and 25.7 μm **(J–L)**.

The charge–discharge cycling performance of the ASSTFLBs with different cathode thicknesses was experimentally studied (Figure 2). The simulated ASSTFLBs were charged and discharged at 0.1 mA. When the LiCoO_2_ thin film is 1.21 μm (Figure 2A), the initial capacity of the ASSTFLBs is 0.12 mAh (Figure 2B) and the corresponding CU is 99.2%. The reversible capacity decreased considerably after 200 cycles, but the Coulombic efficiency remained at a high level of 98.82% at the 500^th^ cycle (Figure 2C). As the thicknesses of the LiCoO_2_ thin films were increased to 2.56 and 10.17 μm (Figures 2D, G), the CU associated with the initial capacity (0.19 and 0.56 mAh) decreased to 74.2% and 55.1%, respectively (Figures 2E, H). Although the reversible capacity became steady after the fast ramping at the beginning stage, the Coulombic efficiency fluctuated (Figures 2F, I). This implies the continuous materials decay during charge and discharge. The ASSTFLB with a 25.7-μm LiCoO_2_ cathode (Figure 2J) cannot be cycled at 10 C due to the limited charge transport kinetics. It performed an initial capacity of 1.69 mAh at 0.05 C, corresponding to a CU of 65.7% (Figure 2K). The Coulombic efficiency at the 20^th^ cycle decreased to 92.92% (Figure 2L). The practically available capacity of ASSTFLBs decreased as the thickness of LiCoO_2_ thin films increased.

**FIGURE 3:**
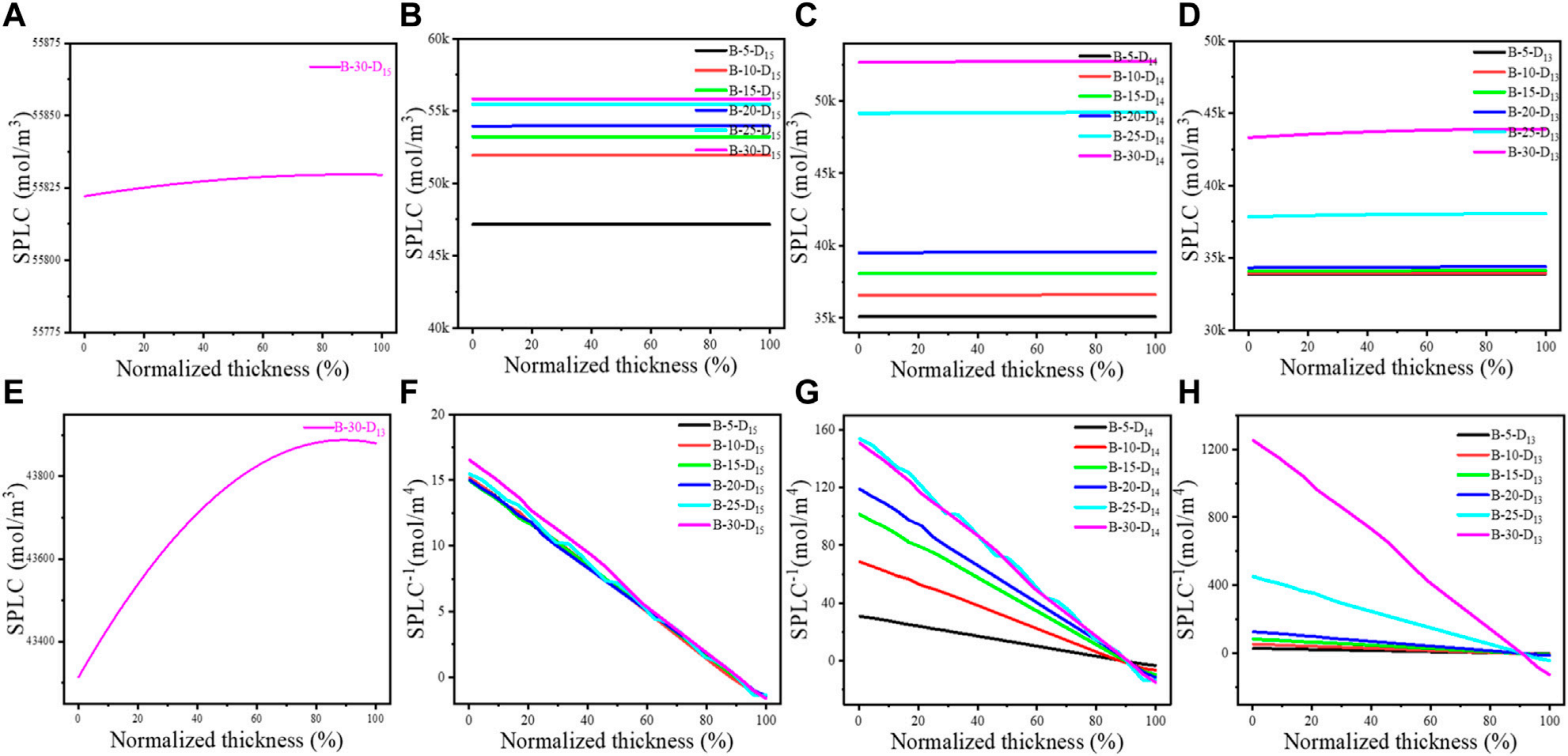
Calculated SPLC **(A–E)** and its gradients **(F–H)** in the LiCoO_2_ cathodes of the modeled ASSTFLBs. For the denotation of the sample labels, please see Supplementary Table S2.

To further understand the effect of Li^+^ conduction on CU, the SPLCs of the LiCoO_2_ thin films in ASSTFLBs after charging, which denotes the degree of Li^+^ extraction from the cathode, were calculated using the presented 1D model. The charging cutoff voltage was set as 4.2 V vs. Li^+^/Li. The initial point of the abscissa refers to the LiCoO_2_-LiPON interface, and the endpoint is the LiCoO_2_-CC interface (Figure 3). Universally, the SPLC at the LiCoO_2_-LiPON interface is lower than that close to the LiCoO_2_-CC interface (Figures 3A–E), which indicates that the Li^+^ is not promptly transported to the LiCoO_2_-LiPON interface because of the limited Li^+^ conduction kinetics. For LiCoO_2_ with a specific Li^+^ diffusivity, the SPLC increases as its thickness increases (Figures 3B–D). This implies the negative effect of limited Li^+^ conductibility on CU in the thicker cathode. In addition, the increased Li^+^ diffusivity would lead to a lower SPLC for the LiCoO_2_ thin films with the same thickness (Figures 3B–D). Moreover, the calculated SPLCs were lower than 35 k mol/m^3^ until the thickness of LiCoO_2_ thin films exceeded 20 μm when Li^+^ diffusivity was assumed to be 1 × 10^−13^ m^2^/s. On the other hand, the SPLC was always higher than 45 k mol/m^3^ when Li^+^ diffusivity was assumed to be 1 × 10^−15^ m^2^/s. In other words, the enhanced Li^+^ conductibility would help extract more Li^+^ from LiCoO_2_ during the charging process.

It is generally believed that the higher Li^+^ diffusivity would result in a more even distribution of SPLC in the LiCoO_2_ thin film. However, the distribution of SPLC of the LiCoO_2_ thin film with the assumed Li^+^ diffusivity of 1 × 10^−15^ m^2^/s was more even than that with the assumed Li^+^ diffusivity of 1 × 10^−13^ m^2^/s when their thicknesses were the same (Figures 3A, E). Furthermore, the LiCoO_2_ thin films with the Li^+^ diffusivity of 1 × 10^−15^ m^2^/s possessed a reduced SPLC gradient compared with their counterparts (Figures 3F–H). This should be attributed to the retention of Li^+^ in the LiCoO_2_ lattice because Li^+^ diffusivity is very low, which is consistent with the calculated SPLC (Figure 3B) and CU (Table 1). The simulated discharge capacity of the first cycle of the simulated ASSTFLBs can be seen in Supplementary Figure S2. Notably, the LiCoO_2_ thin films with the assumed Li^+^ diffusivity of 1 × 10^−13^ m^2^/s also perform the considerable gradients of SPLC, especially if the thickness goes over 20 μm. Therefore, it is necessary to pursue a Li^+^ diffusivity higher than 1 × 10^−13^ m^2^/s for the cathode in ASSLBs.

**TABLE 1:** Calculated initial capacity (IC) and corresponding CU of LiCoO_2_ thin films in ASSTFLBs with different Li^+^ diffusivities (Di) and thicknesses (T).

T	5 μm		10 μm		15 μm		20 μm		25 μm		30 μm	
D_i_	IC (mAh)	CU (%)	IC (mAh)	CU (%)	IC (mAh)	CU (%)	IC (mAh)	CU (%)	IC (mAh)	CU (%)	IC (mAh)	CU (%)
D_13_	0.4173	83.46	0.8500	85	1.2561	83.74	1.6100	80.5	1.996	79.84	0.8522	28.41
D_14_	0.3674	73.48	0.6444	64.44	0.7955	53.03	0.7974	39.87	0.7912	31.65	0.1353	4.51
D_15_	0.0611	12.22	0.0589	5.89	0.0581	3.87	0.0593	2.97	0.0603	2.41	0.0207	0.69

**FIGURE 4:**
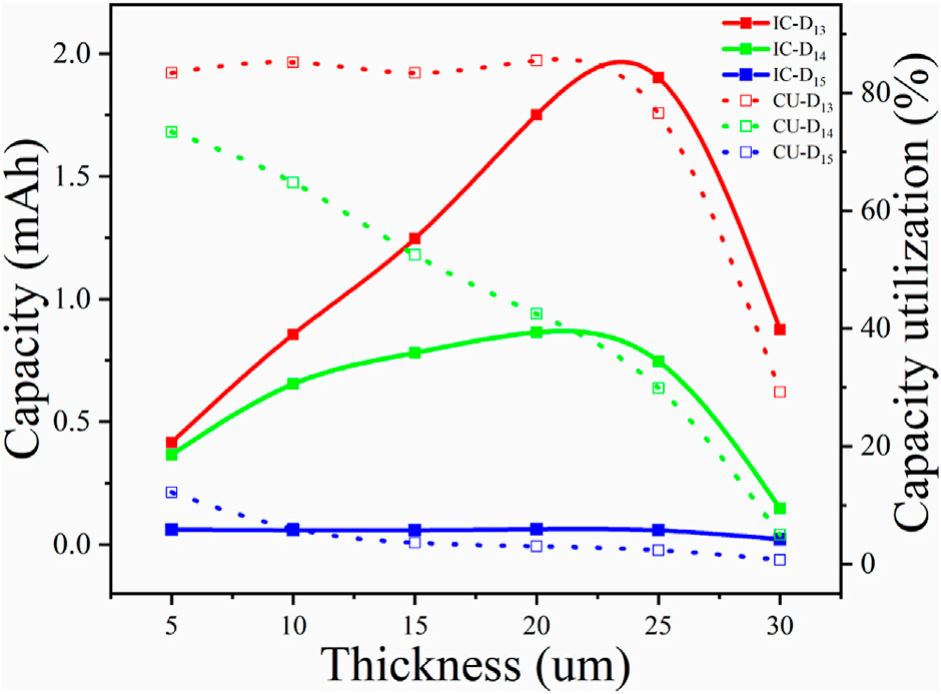
Thickness-dependent capacities and CU of LiCoO_2_ thin films in ASSTFLBs.

The initial capacities and corresponding CU of ASSTFLBs with different Li^+^ diffusivities and thicknesses of LiCoO_2_ thin films were also calculated using the 1D model (Table 1). The charge and discharge current density was set as 0.1 mA, and the cutoff voltage varied from 2.7 to 4.2 V vs. Li^+^/Li. The trend of CU decreasing as the thickness of the LiCoO_2_ thin film increased was consistent with the experiment. In addition, the simulations also showed that the improved Li^+^ diffusivities help achieve higher CU for the thick cathode. The thickness-dependent initial capacity and corresponding CU of LiCoO_2_ thin films in ASSTFLBs are plotted in Figure 4. When the Li^+^ diffusivity of the cathode thin film was lower than 1 × 10^−14^ m^2^/s, the gain of the capacity of ASSTFLBs due to increased thickness is negligible. If the Li^+^ diffusivity of the cathode thin film reached 1 × 10^−13^ m^2^/s, the capacity linearly increased until its thickness exceeded 20 μm. However, the CU remained lower than 85%, which further demonstrated the necessity to enhance the Li conductibility of the cathode for the development of high-performance ASSLBs.

## 4 Conclusion

To summarize, the effects of limited Li^+^ conduction on the capacity utilization of the cathodes in ASSTFLBs were investigated *via* experimentation and simulation. One of the highest area capacities of ASSTFLBs (T 298 k, 1.18 mAh/cm^2^) was achieved *via* increasing the thickness of cathode thin films, but its capacity utilization ratio was far below expectations. The simulations demonstrated that Li^+^ in the cathode of ASSTFLBs cannot be readily transported to the cathode–electrolyte interface during charging, even though the Li^+^ diffusivity was assumed to be one to two orders of magnitude higher than the typical value of pristine LiCoO_2_. Specifically, the capacity utilization ratio of the cathode in ASSTFLBs was always lower than 85%, while the practically available capacity increased considerably as its thickness increased if the Li^+^ diffusivity reached 1 × 10^−13^ m^2^/s and the thickness did not exceed 20 μm. These results emphasize the demand for enhancing the kinetics of charge transport for the following cathode materials studies. Although it remains a challenge to break the bottleneck of Li^+^ conduction in the cathodes of ASSLBs, this study provides feasible analysis methods and quantitative guiding data for future efforts.

## Data Availability

The original contributions presented in the study are included in the article/Supplementary Material; further inquiries can be directed to the corresponding authors.

## References

[B1] Chen R. J. Zhang Y. B. Liu T. Shen Y. Li L.L. Xu B. Q. (2017). All-solid-state lithium battery with high capacity enabled by a new way of composite cathode design. Solid State Ionics 310, 44–49. 10.1016/j.ssi.2017.07.026

[B2] Danilov D. Niessen R. A. H. Notten P. (2011). Modeling All-solid-state li-ion batteries. J. Electrochem Soc. 158 (3), 215–222. 10.1149/1.3521414

[B3] Donders M. E. Arnoldbik W. M. Knoops H. C. M. Kessels W. M. M. Notten P. H. L. (2013). Atomic layer deposition of LiCoO_2_ thin-film electrodes for all-solid-state Li-ion micro-batteries. J. Electrochem Soc. 160 (5), A3066–A3071. 10.1149/2.011305jes

[B4] Doyle M. Fuller T. F. Newman J. (1993). Modeling of galvanostatic charge and discharge of the lithium/polymer/insertion cell. J. Electrochem Soc. 140 (6), 1526–1533. 10.1149/1.2221597

[B5] Duan J. Tang X. Dai H. Yang Y. Wu W. Wei X. (2020). Building safe lithium-ion batteries for electric vehicles: A review. Electrochem Energy R. 3 (1), 1–42. 10.1007/s41918-019-00060-4

[B6] Fuller T. F. Doyle M. Newman J. (1994). Simulation and optimization of the dual lithium ion insertion cell. J. Electrochem Soc. 141 (1), 1–10. 10.1149/1.2054684

[B7] Gao Z. Sun H. Fu L. Ye F. Zhang Y. Luo W. (2018). Promises, Challenges, and recent progress of inorganic solid-state electrolytes for all-solid-state lithium batteries. Adv. Mater 30 (17), 1705702–1705727. 10.1002/adma.201705702 29468745

[B8] Geng Z. Wang S. Lacey M. J. Brandell D. Thiringer T. (2021). Bridging physics-based and equivalent circuit models for lithium-ion batteries. Electrochimica Acta 372, 137829. 10.1016/j.electacta.2021.137829

[B9] Guan M. Huang K. Mou S. W. Jiang C. Z. Pang Y. Xiang A. (2021). Superior ionic conduction in LiAlO_2_ thin-film enabled by triply coordinated nitrogen. AIP Adv. 11 (6), 065310. 10.1063/5.0047625

[B10] Hannan M. A. Hoque M. M. Hussain A. Yusof Y. Ker P. J. (2018). State-of-the-art and energy management system of lithium-ion batteries in electric vehicle applications: Issues and recommendations. Ieee Access 6, 19362–19378. 10.1109/access.2018.2817655

[B11] Judez X. Eshetu G. G. Li C. Rodriguez-Martinez L. M. Zhang H. Armand M. (2018). Opportunities for rechargeable solid-state batteries based on Li-intercalation cathodes. Joule 2 (11), 2208–2224. 10.1016/j.joule.2018.09.008

[B12] Kazemi N. Danilov D. L. Haverkate L. Dudney N. J. Unnikrishnan S. Notten P. H. (2019). Modeling of all-solid-state thin-film Li-ion batteries: Accuracy improvement. Solid State Ionics 334, 111–116. 10.1016/j.ssi.2019.02.003

[B13] Kong L. Xing Y. Pecht M. G. (2018). <italic&gt;*in-situ*&lt;/italic&gt; Observations of Lithium Dendrite Growth. Ieee Access 6, 8387–8393. 10.1109/access.2018.2805281

[B14] Li M. Lu J. Chen Z. Amine K. (2018). 30 years of lithium-ion batteries. Adv. Mater 30 (33), 1800561. 10.1002/adma.201800561 29904941

[B15] Liu G. Lu W. (2017). A model of concurrent lithium dendrite growth, SEI growth, SEI penetration and regrowth. J. Electrochem Soc. 164, A1826–A1833. 10.1149/2.0381709jes

[B16] Liu Q. Su X. Lei D. Qin Y. Wen J. Guo F. (2018). Approaching the capacity limit of lithium cobalt oxide in lithiumion batteries via lanthanum and aluminium doping. Nat. Energy 3 (11), 936–943. 10.1038/s41560-018-0180-6

[B17] Lu P. Liu L. Wang S. Xu J. Peng J. Yan W. (2021). Superior all‐solid‐state batteries enabled by a gas‐phase‐synthesized sulfide electrolyte with ultrahigh moisture stability and ionic conductivity. Adv. Mater 33 (32), 2100921. 10.1002/adma.202100921 34218476

[B18] Ma Y. (2018). Computer simulation of cathode materials for lithium ion and lithium batteries: A review. Energ Environ. Mater 1 (3), 148–173. 10.1002/eem2.12017

[B19] Manthiram A. Yu X. Wang S. (2017). Lithium battery chemistries enabled by solid-state electrolytes. Nat. Rev. Mater 2 (4), 16103–16133. 10.1038/natrevmats.2016.103

[B20] Miao X. Wang H. Sun R. Zhang Z. Li Z. Yin L. W. (2020). Interface engineering of inorganic solid-state electrolytes for high-performance lithium metal batteries. Energ Environ. Sci. 13 (11), 3780–3822. 10.1039/d0ee01435d

[B21] Mou S. W. Huang K. Guan M. Ma X. Chen J. S. Xiang Y. (2021). Reduced energy barrier for Li+ diffusion in LiCoO_2_ via dual doping of Ba and Ga. J. Power Sources 505, 230067–230074. 10.1016/j.jpowsour.2021.230067

[B22] Peng X. Huang K. Song S. P. Wu F. Xiang Y. Zhang X. (2020). Garnet‐polymer composite electrolytes with high Li+ conductivity and transference number via well‐fused grain boundaries in microporous frameworks. ChemElectroChem 7 (11), 2389–2394. 10.1002/celc.202000202

[B23] Raijmakers L. Danilov D. Eichel R. Notten P. (2020). An advanced all-solid-state Li-ion battery model. Electrochimica Acta 330, 135147–135152. 10.1016/j.electacta.2019.135147

[B24] Ramadesigan V. Northrop P. Santhanagopalan S. Braatz R. D. Subramanian V. R. (2012). Modeling and simulation of lithium-ion batteries from a systems engineering perspective. J. Electrochem Soc. 159 (3), 31–45. 10.1149/2.018203jes

[B25] Randau S. Weber D. A. Kötz O. Koerver R. Braun P. Weber A. (2020). Benchmarking the performance of all-solid-state lithium batteries. Nat. Energy 5 (3), 259–270. 10.1038/s41560-020-0565-1

[B26] Song S. P. Peng X. Huang K. Zhang H. Wu F. Xiang Y. (2020). Improved cycling stability of LiCoO_2_ at 4.5 V via surface modification of electrodes with conductive amorphous LLTO thin film. Nanoscale Res. Lett. 15, 110–10. 10.1186/s11671-020-03335-8 32409895 PMC7225228

[B27] Song S. P. Yang C. Jiang C. Z. Wu Y. M. Guo R. Sun H. (2022). Increasing ionic conductivity in Li0.33La0.56TiO3 thin-films via optimization of processing atmosphere and temperature. Rare Met. 41, 179–188. 10.1007/s12598-021-01782-5

[B28] Song S. W. Choi H. Park H. Y. Park G. B. Lee K. C. Lee H. J. (2010). High rate-induced structural changes in thin-film lithium batteries on flexible substrate. J. Power Sources 195 (24), 8275–8279. 10.1016/j.jpowsour.2010.06.113

[B29] Wang Q. Jiang L. Yan Y. Sun J. (2018). Progress of enhancing the safety of lithium ion battery from the electrolyte aspect. Nano Energy 55, 93–114. 10.1016/j.nanoen.2018.10.035

[B30] Wang Q. Ping P. Zhao X. Chu G. Sun J. Chen C. (2012). Thermal runaway caused fire and explosion of lithium ion battery. J. Power Sources 208 (24), 210–224. 10.1016/j.jpowsour.2012.02.038

[B31] Wang T. Li Y. Zhang J. Yan K. (2020). Jaumaux, P. Immunizing lithium metal anodes against dendrite growth using protein molecules to achieve high energy batteries. Nat. Commun. 11 (1), 1–9. 33110084 10.1038/s41467-020-19246-2 PMC7591880

[B32] Wang X. Ding Y. L. Deng Y. P. Chen Z. (2020). Ni-rich/Co-poor layered cathode for automotive li‐ion batteries: Promises and challenges. Adv. Energy Mater 10 (12), 1903864–1903892. 10.1002/aenm.201903864

[B33] Wei C. Chen S. Yu C. Wang R. Luo Q. Chen S. (2023b). Achieving high-performance Li_6. 5_Sb_0. 5_Ge_0. 5_S_5_I-based all-solid-state lithium batteries. Appl. Mater. Today 31, 101770–101778. 10.1016/j.apmt.2023.101770

[B34] Wei C. Yu C. Wang R. Peng L. Chen S. Miao X. (2023a). Sb and O dual doping of Chlorine-rich lithium argyrodite to improve air stability and lithium compatibility for all-solid-state batteries. J. Power Sources 559 (559), 232659–232668. 10.1016/j.jpowsour.2023.232659

[B35] Xia S. Wu X. Zhang Z. Cui Y. Liu W. (2019). Practical Challenges and future perspectives of all-solid-state lithium-metal batteries. Chem 5, 753–785. 10.1016/j.chempr.2018.11.013

[B36] Zhu Y. L. Wu S. Pan Y. Zhang X. Yan Z. Xiang Y. (2020). Reduced energy barrier for Li+ transport across grain boundaries with amorphous domains in LLZO thin films. Nanoscale Res. Lett. 15 (1), 153–158. 10.1186/s11671-020-03378-x 32712882 PMC7382668

[B37] Zubi G. Dufo-López R. Carvalho M. Pasaoglu G. (2018). The lithium-ion battery: State of the art and future perspectives. Renew. Sust. Energy Rev. 89, 292–308. 10.1016/j.rser.2018.03.002

