# Supplementary material for: Effects of Li+ conduction on the capacity utilization of cathodes in all-solid-state lithium batteries
Source: Front Chem. 2023 Apr 21;11:1169896. doi: 10.3389/fchem.2023.1169896 (PMC10160652; doi:10.3389/fchem.2023.1169896)
Supplement: Supplementary file 1 [file DataSheet1.docx]

Effects of limited Li+ conduction on the capacity utilization of cathode in all-solid-sate lithium batteries

Zhiping Wang^1^, Chunzhi Jiang^1^, Shipai Song^1^, Yongmin Wu^2*^, Junsong Chen^1^, Yong Xiang^1,3,4*^, Xiaokun Zhang^1*^

^1^School of Materials and Energy, University of Electronic Science and Technology of China, Chengdu 611731, Sichuan, China.

^2^State Key Laboratory of Space Power-sources Technology, Shanghai Institute of Space Power-sources, Shanghai 200245, China

^3^Advanced Energy Research Institute, University of Electronic Science and Technology of China, Chengdu 611731, Sichuan, China

^4^Sichuan Provincial Engineering Research Center of Flexible Display Material Genome, University of Electronic Science and Technology of China, Chengdu, Sichuan, 611731, China

†These authors contribute equally to this work.

*** Correspondence:**Xiaokun Zhang, Yongmin Wu
zxk@uestc.edu.cn
Yongmin Wu
wuym2014@126.com
Yong Xiang
xyg@uestc.edu.cn


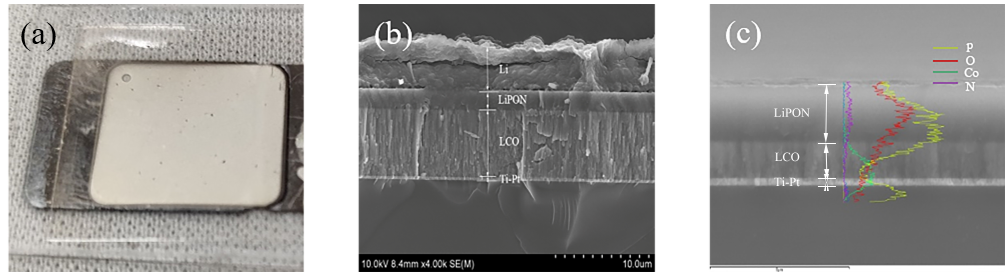


Fig. S1. A typical photo (a), cross-sectional SEM image (b), and EDS depth profile (c) of the presented ASSTFLBs.

Table S1 Simulation parameters of the ASSTFLBs model

| **LCO parameters** | **Numerical value** |
| --- | --- |
| Conductivity（mS/cm） | 1.13 |
| Diffusion coefficient(m^2^/s) | 1×10^-13^, 1×10^-14^,1×10^-15^ |
| Solid phase lithium concentration(mol/m^3^) | 56194 |
| Density(g/cm^3^) | 4.69 |
| SOC | 0.43-1 |
| Thickness（um） | 5,10,15,20,25,30 |
| **LiPON parameters** |  |
| Conductivity（mS/cm） | 9.05×10^-6^ |
| Diffusion coefficient(m^2^/s) | 7.5×10^-11^ |
| Transfer number | 0.363 |
| Thickness（um） | 2 |
| **Li parameters** |  |
| Current exchange density（A/m^2^） | 100 |
| Anode transfer coefficient | 0.5 |
| Thickness（um） | 2 |

Table S2**.** The denotation of the sample labels

| **D(**m^2^/s**)**  **T(μm)** | D_13_=**1×10^-13^** (m^2^/s) | D_14_=**1×10^-14^** (m^2^/s) | D_15_=**1×10^-15^** (m^2^/s) |
| --- | --- | --- | --- |
| 5 | B-5-D_13_ | B-5-D_14_ | B-5-D_15_ |
| 10 | B-10-D_13_ | B-10-D_14_ | B-10-D_15_ |
| 15 | B-15-D_13_ | B-15-D_14_ | B-15-D_15_ |
| 20 | B-20-D_13_ | B-20-D_14_ | B-20-D_15_ |
| 25 | B-25-D_13_ | B-25-D_14_ | B-25-D_15_ |
| 30 | B-30-D_13_ | B-30-D_14_ | B-30-D_15_ |

Table S3**.** The denotation of the sample labels

| T(μm) | Theoretical capacity(mAh/cm^2^) |
| --- | --- |
| 1.21 | 0.085 |
| 2.56 | 0.179 |
| 10.17 | 0.711 |
| 25.70 | 1.796 |

Table S4**.** The denotation of the sample labels

| ASSTFLBs | Capacity | Refs. |
| --- | --- | --- |
| LiCoO_2_/LiPON/Li | 69 μAh/cm^2^ |  |
| LiCoO_2_/Li_2_O-V_2_O_5_-SiO_2_/SnO | 9.0 μAh/cm^2^ |  |
| LiCoO_2_/LiPON/Si | 16 μAh/cm^2^ |  |
| TiO_2_/LiPON/Li | 20 μAh/cm^2^ |  |
|  |  |  |


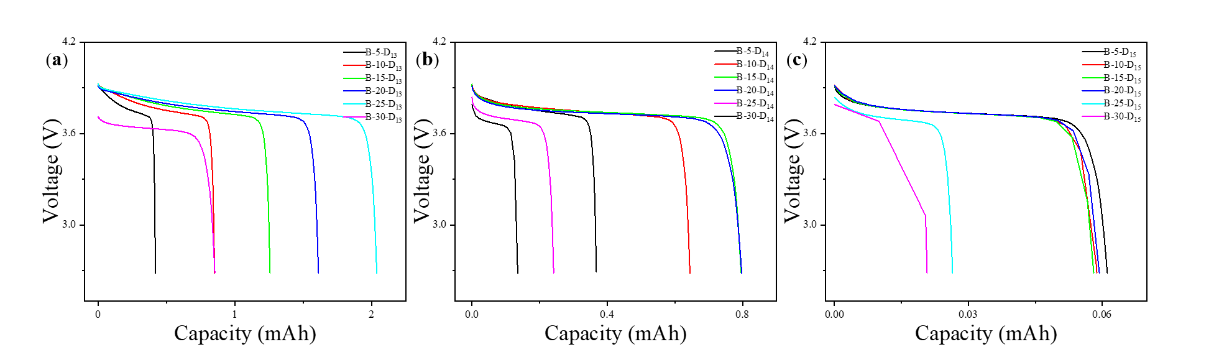


Fig. S2. The initial discharge curves of LCO with different thicknesses and assumed Li^+^ diffusivities: (a) 1×10^-13^ m^2^/s, (b) 1×10^-14^ m^2^/s, (c) 1×10^-15^ m^2^/s.
